# Feline Panleukopenia Virus in a Marsican Brown Bear and Crested Porcupine, Italy, 2022–2023

**DOI:** 10.3201/eid3012.240505

**Published:** 2024-12

**Authors:** Georgia Diakoudi, Gianvito Lanave, Shadia Berjaoui, Costantina Desario, Giovanni Di Teodoro, Violetta Iris Vasinioti, Francesco Pellegrini, Sabrina V.P. Defourny, Stefania Salucci, Antonio Cocco, Alessio Lorusso, Vito Martella, Nicola Decaro

**Affiliations:** University of Bari Aldo Moro, Bari, Italy (G. Diakoudi, G. Lanave, C. Desario, V.I. Vasinioti, F. Pellegrini, V. Martella, N. Decaro); Istituto Zooprofilattico Sperimentale di Abruzzo e Molise ‘Giuseppe Caporale,’ Teramo, Italy (S. Berjaoui, G. Di Teodoro, S.V.P. Defourny, S. Salucci, A. Cocco, A. Lorusso); University of Veterinary Medicine, Budapest, Hungary (V. Martella)

**Keywords:** Feline panleukopenia virus, canine parvovirus, Protoparvovirus carnivoran1, brown bear, porcupine, sequence analysis, molecular characterization, viruses, Italy

## Abstract

The virus species *Protoparvovirus carnivoran 1* encompasses pathogens that infect both domestic and wild carnivores, including feline panleukopenia virus. We identified and characterized feline panleukopenia virus strains in a Marsican brown bear (*Ursus arctos marsicanus*) and a crested porcupine (*Hystrix cristata*) in Italy, extending the known host range of this virus.

Parvoviruses (genus *Protoparvovirus*, family Parvoviridae) are small, nonenveloped, single-stranded DNA viruses ≈4.5–5.5-kb long. Their linear DNA genome contains 2 major open reading frames encoding for 2 nonstructural proteins (1 and 2) and 2 capsid proteins (VP1 and VP2). Feline panleukopenia virus (FPV) and the closely related canine parvovirus (CPV or CPV-2), included in the species *Protoparvovirus carnivoran 1*, are highly contagious pathogens that cause acute and often fatal diseases in domestic and wild felids and canids (*1*).

FPV and CPV are closely related genetically and antigenically but differ in their host range and pathogenicity. The biological differences are determined by a few amino acid mutations in the VP2 capsid protein that modulate the ability to bind to cellular transferrin receptor type 1 (*2*). Parvoviruses have long been considered to be species specific; however, FPV and CPV-2 variants (i.e., 2a, 2b, and 2c) have been reported in a wide variety of wild carnivores. Of note, some species (e.g., cats, foxes, badgers, and giant pandas) may be infected by both FPV and CPV strains (*3*). We conducted a molecular investigation for *Protoparvovirus carnivoran 1* viruses in wildlife in Italy.

## The Study

During January 2022–May 2023, we collected tissue samples by convenience sampling from 89 animals: 44 wolves (*Canis lupus*), 26 red foxes (*Vulpes vulpes*), 15 European badgers (*Meles meles*), 2 stone martes (*Martes foina*), 1 Marsican brown bear (*Ursus arctos marsicanus*), and 1 crested porcupine (*Hystrix cristata*). All animals were found already dead in the regions of Abruzzo and Molise, Italy, and sample collection was conducted during routine necropsy procedures at Istituto Zooprofilattico Sperimentale of Abruzzo and Molise ‘Giuseppe Caporale.’ All tissues were temporarily stored at −20°C before being transported by cold chain to the Infectious Diseases Unit of the Department of Veterinary Medicine, University of Bari, Bari, Italy.

We homogenized the tissues (10% wt/vol) in Dulbecco’s modified Eagle medium and extracted viral DNA from the supernatant of the homogenates of all samples by using the IndiSpin Pathogen Kit (Indical Bioscience GmbH, https://www.indical.com), according to the manufacturer’s instructions. We used quantitative PCR (qPCR) to screen DNA extracts for the presence of CPV/FPV DNA (*4*) and further characterized positive samples by qPCR based on minor groove binder able to differentiate CPV types 2a/2b and 2b/2c and CPV/FPV, as described previously (*4*).

Overall, 52 (58.4%) of 89 samples tested positive in the qPCR screening for CPV/FPV DNA (Table 1) with a geometric mean value of 8.91 × 10^3^ copies of parvovirus DNA/10 μL of template (range 8.48 × 10^0^ to 5.71 × 10^6^ DNA copies/10 μL of template), including the samples from the Marsican brown bear and the crested porcupine. We characterized the bear and porcupine viruses as FPV by using the minor groove binder qPCR with viral loads of 8.40 × 10^5^ (bear) and 4.43 ×10^5^ (porcupine) DNA copies/10 μL of template.

**Table 1:** Results of testing for *Protoparvovirus carnivoran1* viruses in wild animals, Abruzzo and Molise regions, Italy, January 2022–May 2023*

Animal species	No. animals tested	No. animals positive				Total no. (%) samples positive, tissue type
		FPV	CPV-2a	CPV-2b	CPV-2c	
Wolf (*Canis lupus*)	44	7	4	11	8	30 (68.2), I = 27, S = 3
Red fox (*Vulpes vulpes*)	26	3	7	0	2	12 (46.2), I = 12
European badger (*Meles meles*)	15	5	0	2	0	7 (46.7), I = 6, MLN = 1
Stone martens (*Martes foina*)	2	1	0	0	0	1 (50), I = 1
Marsican brown bear (*Ursus arctos marsicanus*)	1	1	0	0	0	1 (100), I = 1
Crested porcupine (*Hystrix cristata*)	1	1	0	0	0	1 (100), MLN = 1
Total	89	18	11	13	10	52 (58.4), I = 47, S = 3, MLN = 2

*CPV, canine parvovirus; FPV, feline panleukopenia virus; I, intestine; MLN, mesenteric lymph node; S, spleen.

To acquire the complete viral genome sequence of the FPV strains from the 2 animals, we designed 2 multiplex PCR protocols amplifying 15 PCR-tiling amplicons of 388–511 bp (Table 2), following an ARTIC-like strategy (*5*). We designed primer pairs based on the consensus sequence of *Protoparvovirus carnivoran1* genomes recovered from GenBank. We performed PCR by using TaKaRa La Taq polymerase (Takara Bio Europe, https://www.takarabio.com). We used 500 ng of equimolar pooled PCR products for each FPV-positive sample as the input to the libraries prepared by using Ligation Sequencing Kit (SQK-LSK110; Oxford Nanopore Technologies, https://nanoporetech.com), according to the manufacturer’s guidelines. We sequenced the libraries independently by using flongle flowcell FLO-FLG001, R9.4.1, adapted in a MinION Mk1C platform (Oxford Nanopore Technologies) for 24 hours each. We subjected FastQ MinION files to quality control, trimming, and reference assembly by using Minimap2 plugin implemented in Geneious Prime software v.2021.2.2 (Biomatters Ltd., https://www.geneious.com).

**Table 2:** Oligonucleotides used for the multiplex PCR protocols based on ARTIC-like strategy used in study of feline panleukopenia virus in wild animals, Abruzzo and Molise, Italy, January 2022–May 2023*

Primer	Sequence, 5′ → 3′	Amplicon size, bp
CPV1_left	AATGATAGGCGGTTTGTGTGTTT	396
CPV1_right	CAACTTCCGATTCCCAGTCCAT	396
CPV2_left	CCAATTCAAAATGAAGAGCTAACATCTT	410
CPV2_right	GTCACCCATTCACTATCTTCTGCA	410
CPV3_left	TGGAGTAGATGGTTGGTGACTCT	416
CPV3_right	ACCAAGTCCCGCAAAGTACATT	416
CPV4_left	AGCACACTTTACACTGAACAAATGA	401
CPV4_right	GCTATAGCGTGACAAACTTTAATCCA	401
CPV5_left	AGCAAGAACAAAAACAGCATTTGAA	402
CPV5_right	TGATCAATTCTAATTGTTTGTCCAGAACA	402
CPV6_left	AAAAATTTAATTTGGATTGAAGAAGCTGGT	407
CPV6_right	CCTTCTTGTATTTTAGGCTCCGC	407
CPV7_left	TGAATCAACCATGGCTAACTATACACA	393
CPV7_right	GGAGGTGCCATCGTACCTTAATC	393
CPV8_left	TGGTCCGAAATAGAGGCAGACC	393
CPV8_right	CCCCCAATCTTTAGCGTCCTTA	393
CPV9_left	GCTGCTTATCTTCGCTCTGGTA	412
CPV9_right	AAATCCCCACACCCCCAGAA	412
CPV10_left	GCACCAATGAGTGATGGAGCA	410
CPV10_right	TCACTCATAGTATTAACAATTAGTTGCCA	410
CPV11_left	TGCACAAATTGTAACACCTTGGT	388
CPV11_right	ATTTGTTGGTGTGCCACTAGTTC	388
CPV12_left	ACCAACCATACCAACTCCATGG	411
CPV12_right	TAACCAACCTCAGCTGGTCTCA	411
CPV13_left	GGAGTTCAACAAGATAAAAGACGTGG	396
CPV13_right	ACCTCCAATTGGATCTGTTGGT	396
CPV14_left	TCCAGAAGGAGATTGGATTCAAAATATT	411
CPV14_right	TTCCAAGTATGAGAGGCTCTTAGTT	411
CPV15_left	TTGATACTGACTTAAAACCAAGACTTCA	511
CPV15_right	ACAGTTATTGTATACCATATAACAAACCTTCT	511

*ARTIC-like strategy as shown in (*5*). CPV, canine parvovirus.

We generated the complete coding regions of the genomes for FPV strains ITA/2023/bear/74 (submitted to GenBank under accession no. OR602717) and ITA/2023/hystrix/213 (submitted to GenBank under accession no. OR602718). Sequence alignment of the complete VP2 genomic region revealed 2 aa mutations, 103-Val to Ala in both capsid sequences and 232-Val to Ile in the porcupine capsid, which are 2 key residues defining the biological properties and host range of CPV and FPV (*2*). VP2 residues 103- and 232- are Val in reference FPV strains, and they are replaced by Ala and Ile in CPV-2 strains. Phylogenetic analysis of the complete amino acid sequence of the VP2 gene indicated that the 2 FPV strains ITA/2023/bear/74 and ITA/2023/hystrix/213 were segregated together but separately from other FPV and CPV strains. Of note, sequencing of the FPV strains detected in other species (wolf and badger) evidenced circulation of >3 different FPV subclades/strains in the sampled animals (Figure). Also, based on our results and the geographic areas where the samples were collected, we can hypothesize that there could be an epidemiologic connection via either direct/indirect contact between porcupines and bears or other cohabiting wild animal species.

**Figure:**
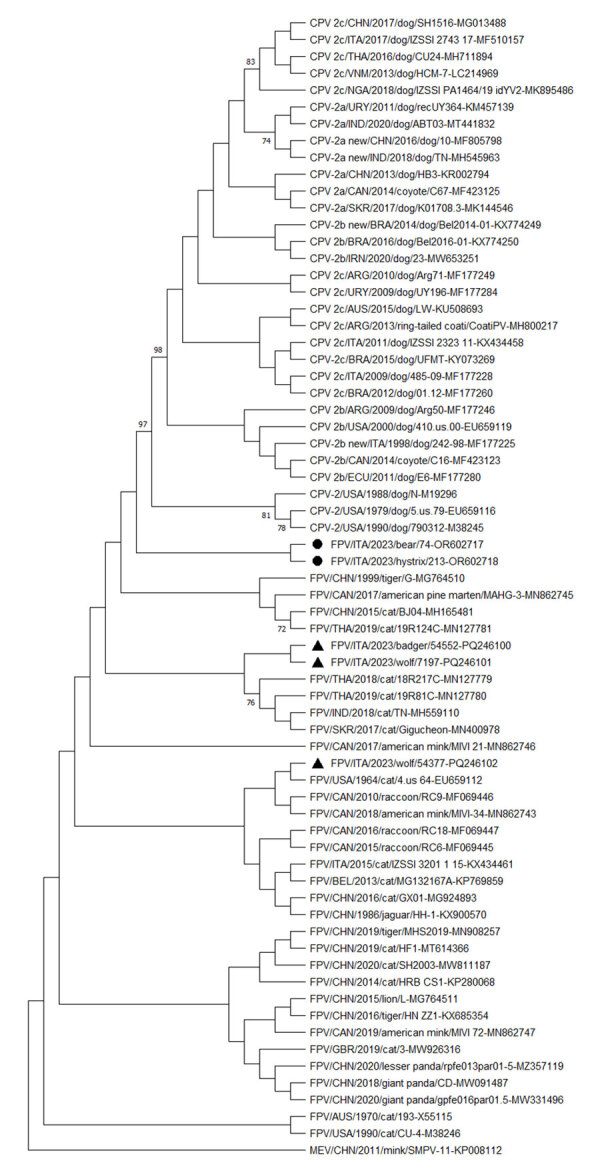
Neighbor-joining capsid protein 2 (VP2)–based phylogenetic tree of *Protoparvovirus carnivoran 1* from study of feline panleukopenia virus (FPV) in wild animals, Abruzzo and Molise, Italy, January 2022–May 2023. The tree was elaborated by using a 584-aa long alignment of the VP2 sequences of the FPV strains generated in the study and the cognate sequences of *Protoparvovirus carnivoran 1* strains retrieved from GenBank. The tree was constructed by using MEGA X version 10.0.5 software (https://www.megasoftware.net) and the maximum-likelihood method, the Jones-Taylor-Thornton substitution model, and bootstrapping up to 1,000 replicates. Bootstrap values >70% are shown. Black bullets indicate FPV strains from a Marsican brown bear (ΙΤΑ/2023/bear/74 (GenBank accession no. OR602717) and a crested porcupine ITA/2023/hystrix/213 (accession no. OR602718). The black triangles indicate FPV strains from other wildlife animals in the same study: ITA/2023/badger/54552 (accession no. PQ246100), ΙΤΑ/2023/wolf/7197 (accession no. PQ246101), and ΙΤΑ/2023/wolf/54377 (accession no. PQ246102). MEV (accession no. KP008112) was used as an outgroup. Scale bar indicates number of amino acid substitutions per site. CPV, canine parovirus; MEV, mink enteritis virus; FPV, feline panleukopenia virus.

Immunofluorescence analysis of the mesenteric lymph node tissues of the crested porcupine detected parvovirus antigens (Appendix Figure). However, immunofluorescence analysis conducted on the bear samples did not allow correct interpretation because of advanced postmortem degradation of the intestinal tissue.

## Conclusions

Serologic studies performed for several bear species (members of the family Ursidae) have shown evidence of exposure to FPV/CPV (*6*–*8*). Two serologic studies involving Marsican brown bears reported seroprevalences as high as 40% (*7*) and 100% (*8*) for FPV/CPV, although those tests do not differentiate between CPV-2 and FPV. The possibility that FPV and CPV can infect bears has also been confirmed by detection and molecular characterization of FPV and CPV-2a from giant pandas in China (*9*,*10*). In both of those instances, the VP2-coding region presented unusual amino acid mutations (i.e., 299-Gly to Glu in the FPV virus and 370-Arg to Gln in the CPV-2a strain). Whether those unique amino acid variations affected the host range of the parvovirus strains remains unknown.

A case report from 1984 in Canada described a suspected parvovirus infection in porcupines that exhibited clinical signs suggestive of parvoviral infection (*11*). However, electron microscopy, serologic tests, and virologic investigations did not confirm the etiology.

Recently, several new parvoviruses (e.g., bocaparvoviruses and bufaviruses) have been discovered in domestic and wild animals (*12*). CPV and FPV seem to be endemic to wildlife populations and have shown continuous evolution over the years; CPV/FPV-like strains have emerged with a broadened host range (*3*,*13*). Domestic pets are regarded as a source of FPV and CPV strains for wildlife carnivores through spillover infections (*3*,*14*). However, wildlife hosts may also serve as virus reservoirs and sources of infection for the domestic animal population in a perpetual cycle involving 2-way transmission among susceptible hosts. In our study, prevalence of CPV and FPV virus in wildlife was higher than expected (*3*,*15*). Genome sequencing of FPV strains detected from nonconventional FPV/CPV hosts, the Marsican brown bear and the crested porcupine, revealed amino acid mutations in key residues controlling host range and receptor affinity. Our detection of FPV strains in a Marsican brown bear and in a crested porcupine in Italy extends the known host range of this virus.

Appendix Additional results from study of feline panleukopenia virus in wild animals, Abruzzo and Molise, Italy, January 2022–May 2023.
