# Supplementary material for: Feline Panleukopenia Virus in a Marsican Brown Bear and Crested Porcupine, Italy, 2022–2023
Source: Emerg Infect Dis. 2024 Dec;30(12):2655–9. doi: 10.3201/eid3012.240505 (PMC11616640; doi:10.3201/eid3012.240505)
Supplement: Appendix — Additional results from study of feline panleukopenia virus in wild animals, Abruzzo and Molise, Italy, January 2022–May 2023. [file 24-0505-Techapp-s1.pdf]

Article DOI: https://doi.org/10.3201/eid3012.240505

EID cannot ensure accessibility for supplementary materials supplied by authors. Readers who have difficulty accessing supplementary content should contact the authors for assistance.

Feline Panleukopenia Virus in a Marsican Brown Bear and Crested Porcupine, 2022–2023

Appendix

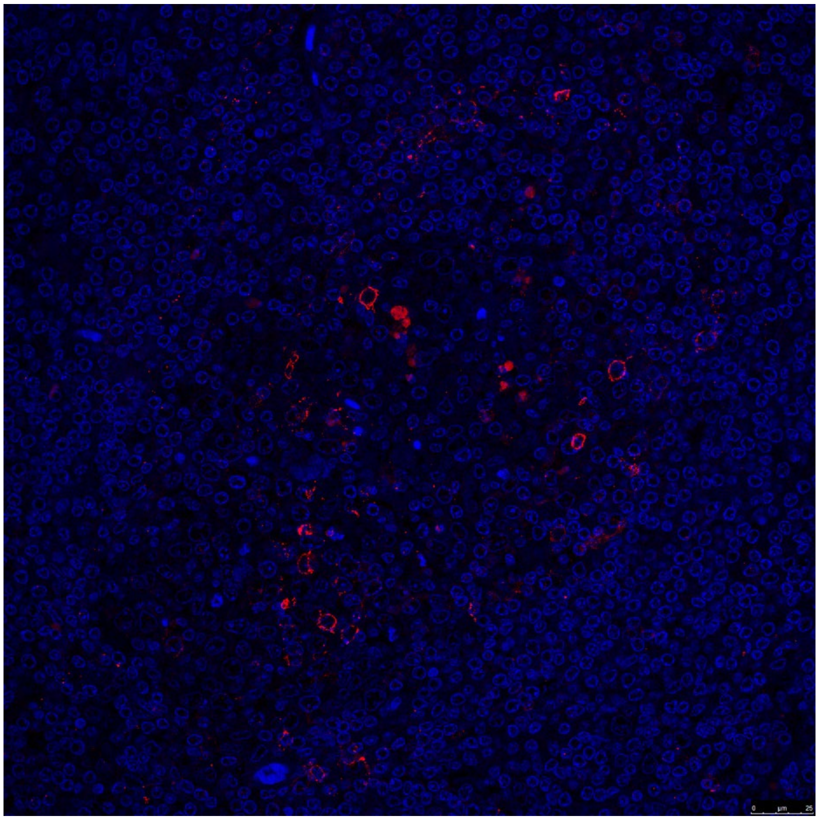

Appendix Figure. Immunofluorescence in mesenteric lymph node of a crested porcupine (*Hystrix cristata*) for CPV/FPV (*Protoparvovirus carnivoran1*). Formalin fixed and paraffin embedded tissue sections were incubated with a primary antibody anti-CPV (1:50 dilution, rabbit polyclonal antibody) that cross reacts with FPV, followed by incubation with a fluorescein isothiocyanate-conjugated goat anti-rabbit IgG (1:100, ThermoFisher Scientific, https://www.thermofisher.com) used as secondary antibody. CPV-antigens (red color) were detected in cells residing in a germinal center of a lymphoid follicle. Nuclei are counterstained with 4′,6-Diamidino-2-Phenylindole (DAPI). Control sections of mesenteric lymph node tissues from other crested porcupines were negative. Scale bar indicates 25 μm.
